# RNA Sequencing Reveals the Differentially Expressed circRNAs between Stable and Unstable Carotid Atherosclerotic Plaques

**DOI:** 10.1155/2023/7006749

**Published:** 2023-03-27

**Authors:** Xueguang Lin, Ying Deng, Lujuan Ye, Bo Chen, Jindong Tong, Weijun Shi, Bo Wang, Bo Yu, Jingdong Tang

**Affiliations:** ^1^Department of Vascular Surgery, Shanghai Pudong Hospital, Fudan University Pudong Medical Center, Shanghai 201399, China; ^2^Fudan Zhangjiang Institute, Shanghai 201203, China; ^3^Shanghai Key Laboratory of Vascular Lesions Regulation and Remodeling, Shanghai, China; ^4^Department of Vascular Surgery, Ganzhou People's Hospital, Ganzhou, Jiangxi 341000, China

## Abstract

**Objective:**

This study aimed to identify circular RNA profiles (circRNAs) via high-throughput RNA sequencing and distinguish the differentially expressed (DE) circRNAs between stable and unstable plaques.

**Methods:**

RNA sequencing was performed on unstable and stable carotid plaque samples obtained from patients with carotid artery stenosis. DE circRNAs were screened, and six DE circRNAs were verified using quantitative real-time PCR (qRT-PCR). Functional evaluation of the DE circRNAs was conducted via Gene Ontology (GO) and Kyoto Encyclopedia of Genes and Genomes (KEGG) pathway enrichment analyses.

**Results:**

We screened 344 DE circRNAs in unstable plaques, consisting of 342 upregulated and 2 downregulated circRNAs. GO analysis showed that the host genes of the upregulated circRNAs were related to ER to Golgi transport vesicle membrane, endocytic vesicle membrane, and Ran GTPase binding. KEGG analysis revealed that the host genes of the upregulated circRNAs were primarily associated with protein processing in endoplasmic reticulum, lysine degradation, homologous recombination, epithelial cell signaling in *Helicobacter pylori* infection, and yersinia infection. The results of qRT-PCR verified three upregulated DE circRNAs and two downregulated DE circRNAs in unstable plaques.

**Conclusion:**

Hsa-circ-0001523, hsa-circ-0008950, hsa-circ-0000571, hsa-circ-0001946, and hsa-circ-0000745 may be involved in regulating the stability of atherosclerotic plaques and serves as a therapeutic target for unstable plaques.

## 1. Introduction

Atherosclerosis is a chronic systemic inflammatory disease of the arterial wall and is one of the foremost reasons for the morbidity and mortality of cardiovascular diseases worldwide. The distinguishing feature of this disease is the formation of atherosclerotic plaques in the arteries, which are attributed to lipid accumulation, inflammatory cell infiltration, cell apoptosis, and increased extracellular matrix secretion [1]. Different from stable plaques, vulnerable/unstable plaques have an active inflammatory response that contributes to the thinning of the fibrous cap and to plaque rupture, which is a major cause of fatal cardiovascular diseases [2, 3]. Studies have demonstrated that the dysregulation of genes that play critical roles in the stability of atherosclerotic plaques predisposes them to rupture and thrombosis formation [4, 5]. Therefore, identifying the dysregulation of active molecules that affect the instability of plaques is important for the diagnosis, treatment, and prognosis of unstable atherosclerotic plaques.

Circular RNAs (circRNAs) are characteristic of unique covalently closed loops and constitute a new family of endogenous noncoding RNAs (ncRNAs). Owing to their regulatory activity in many biological processes, circRNAs have been recently recognized as potential diagnostic and therapeutic targets in various diseases [6, 7]. As reported in the literature, biological activities associated with the presence of atherosclerotic plaques, including inflammation, apoptosis, and lipid metabolism, are activated or deactivated by circRNAs [8, 9]. There is more direct evidence that circRNAs are associated with atherosclerotic stroke [10, 11] and affect atherosclerotic plaque stability [5]. However, there is a lack of comprehensive understanding of the influence of circRNAs in plaque stability.

In this study, circRNAs expression patterns in human stable and unstable plaques were analyzed using RNA sequencing, and the differentially expressed (DE) circRNAs and their potential functions in unstable plaques were further explored. Our findings revealed a comprehensive understanding of the circRNA expression pattern associated with atherosclerotic plaque instability.

## 2. Materials and Methods

### 2.1. Samples from Patients with Carotid Stenosis

Atherosclerotic plaques were collected from patients with carotid stenosis who underwent carotid endarterectomy in our hospital. Plaques were classified based on criteria from the American Heart Association [12] by two independent investigators. Plaques in types I–VIII were categorized as stable, and plaques in types IV–VI as unstable. In this study, three patients with unstable plaques were enrolled, and three patients with stable plaques were enrolled, with their clinical information as shown in Table 1. A total of three stable atherosclerotic plaque samples and three unstable atherosclerotic plaque samples were obtained and placed immediately in liquid nitrogen. Plaques were stored in a −80°C fridge until RNA sequencing and quantitative real-time PCR (qRT-PCR) assay were performed. Our research protocols have been approved by the Ethics Committee of Shanghai Pudong Hospital. All participants provided informed consent before the study procedure began.

### 2.2. Total RNA Extraction and Library Construction

The TRIzol reagent (Thermo, USA) was employed to extract total RNA from the stable and unstable groups. RNA samples were strictly controlled in three aspects: I. RNA integrity and presence of DNA contamination detected using agarose gel electrophoresis; II. RNA purity detected using a NanoDrop spectrophotometer (Thermo Scientific, USA); III. accurate detection of RNA integrity via an Agilent 2100 bioanalyzer (Agilent Technologies, USA).

To perform library sequencing, ribosomal RNA was removed from the total RNA using a Ribo-Zero Magnetic Kit (EpiCentre, Beijing, China). The generated RNAs were interrupted randomly and then used for library preparation with the Illumina Truseq™ RNA Sample Prep Kit with Ribo-Zero (Illumina, USA). Synthesis of the first-strand cDNA was catalyzed using SuperScript II reverse transcriptase, and the second-strand cDNA synthesis was catalyzed via DNA polymerase I and RNase H. The generated cDNAs were adenylated at the 3′ end and ligated using Illumina PE adapter oligonucleotides. The library was obtained using DNA fragments enriched via 15 cycles of PCR with Illumina PCR Primer Cocktail.

### 2.3. RNA Sequencing

All the samples were sequenced via sequencing-by-synthesis technology on a NovaSeq 6000 platform. The produced raw data in FASTQ format were subjected to qualitative filtering to remove low-quality reads (10% of reads containing N; small fragments less than 25 bp in length after quality clip) using Cutadapt (v1.15) software. The generated clean data were mapped to the reference genome (GRCh38) on HISAT2 v2.0.5 software, and circRNAs were annotated using CIRI2 software.

### 2.4. DE circRNA Identification

The intersample Pearson correlation of gene expression levels was analyzed to evaluate the rationality of sample selection. For DE circRNA analysis, the read count of circRNAs (back-spliced junction read) was normalized, and the resulting *P* values were adjusted to control the false discovery rate (FDR). DE circRNAs between the stable and unstable plaque tissues were analyzed using DESeq2 based on the screening criteria of |log_2_ fold change| ≥ 1 and FDR < 0.05 [13].

### 2.5. Gene Ontology (GO) Terms and Kyoto Encyclopedia of Genes and Genomes (KEGG) Pathway Analysis of the Upregulated circRNAs

The potential functions of the upregulated circRNAs in unstable plaques were predicated by the annotation of GO terms and analysis of KEGG pathways using clusterProfiler in R package. There were three GO categories for enriched host genes, namely, biological process (BP), cell component (CC), and molecular function (MF) [14]. *P* value <0.05 represented the significance of enriched terms or KEGG pathways.

### 2.6. qRT-PCR

Six DE circRNAs, i.e., four upregulated circRNAs and two downregulated circRNAs, were selected for qRT-PCR verification (*n* = 3 for each group). Briefly, total RNA was obtained from three stable and three unstable homogenized plaque tissues using RNAiso Plus (TAKARA 9190; Takara Biomedical Technology (Beijing) Co., Ltd, China). The purified RNA precipitates were dissolved in DEPC H_2_O, and a spectrophotometer was used to measure the purity and concentration of the isolated total RNA. Subsequently, total RNA (1000 ng) was reverse transcribed into the cDNA (10 *μ*L) of the reaction system using PrimeScript RT Master Mix (TAKARA RR036A; Takara Biomedical Technology (Beijing) Co., Ltd., China). The produced cDNA was amplified using fluorescent qRT-PCR using Power SYBR Green PCR Master Mix (Thermo 4367659; USA), following the manufacturer's protocol. The amplification conditions were 95.0°C for 10 min, followed by 40 cycles of 95.0°C for 15 s and 60.0°C for 60 s. The sequences of the used primers are listed in Table 2. The relative expression levels of the related DE circRNAs were determined using the 2^−ΔΔCT^ method, with GAPDH as the internal control.

### 2.7. Statistical Analysis

The data were presented as mean ± standard deviation. A comparison between the two groups was conducted via Student's *t* test in Graphpad Prism 5 software (USA). A *P* value <0.05 was considered statistically significant.

## 3. Results

### 3.1. circRNA Profiles in Plaques by RNA Sequencing and Identification of DE circRNAs

**Figure 1:**
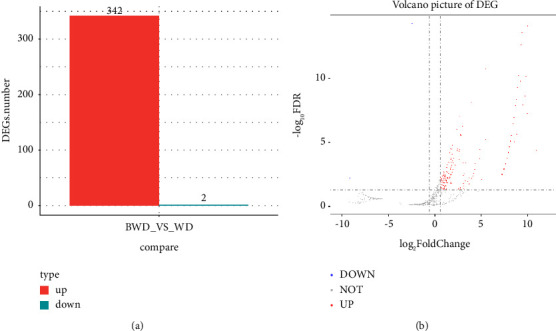
Profiles of circRNAs and their differential expressions comparing stable and unstable plaques. (a) The histogram displays 342 differentially expressed upregulated circRNAs and 2 downregulated circRNAs in unstable plaques, as compared with those of stable plaques. (b) The volcano plot shows the differentially expressed (DE) circRNAs in unstable plaques. The horizontal dashed-dotted line shows the adjusted *P* value of 0.05, and the vertical dashed-dotted lines represent the twofold change in genes. For the unstable plaques, upregulated circRNAs are indicated by red points, and downregulated circRNAs are displayed as blue points.

A total of 2450 circRNAs were annotated by RNA sequencing in 3 stable plaques and 3 unstable plaques. Based on the |log_2_ FC| ≥  1 and FDR < 0.05 criteria and comparing the stable and unstable plaques, we identified 344 DE circRNAs in the unstable plaques, comprising 342 upregulated and 2 downregulated DE circRNAs (Figure 1(a)). The most evident DE circRNAs are shown in volcano plots in Figure 1(b).  Table 3 displays the top 15 upregulated circRNAs from the unstable plaques.

### 3.2. GO Terms and KEGG Pathway Enrichment of the Upregulated DE circRNAs

**Figure 2:**
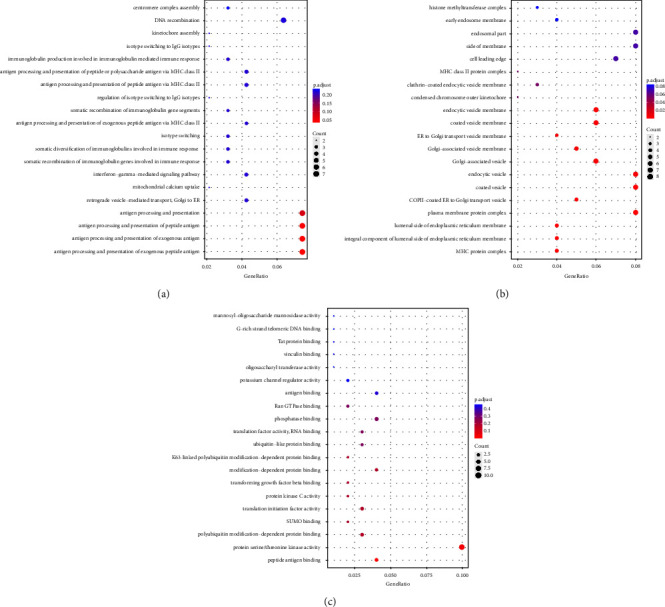
GO annotation of the host genes of the upregulated circRNAs. The bar plot displays the gene numbers of the top 20 GO terms with significant enrichment in the (a) biological process, (b) cellular component, and (c) molecular function categories.

**Figure 3:**
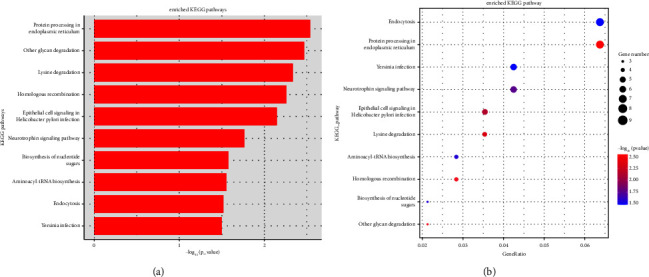
KEGG enrichment for the host genes of the upregulated circRNAs. (a) The top 10 significantly enriched KEGG pathways of upregulated circRNAs are shown by the bar plot. The horizontal axis represents the enrichment scores (−log_10_[*P* value]). (b) Bulb map displays the enriched KEGG pathways of the host genes of upregulated circRNAs. The horizontal axis represents the enrichment degree of the upregulated genes. The vertical axis displays the different terms of the top 10 pathways enriched by upregulated genes. The number of enriched host genes of DE circRNAs is expressed by different node sizes. A color scale shows the different *P* values.

Given that most DE circRNAs were upregulated, we predicted the function of the upregulated DE circRNAs based on the GO and KEGG databases. GO enrichment analysis focused on the three categories (BP, CC, and MF). According to the enriched host gene of upregulated circRNAs, we ranked the top 20 GO terms in the three categories in Figure 2. Notably, the most enriched GO terms in each of the three categories were isotype switching to IgG isotypes, mitochondrial calcium uptake, antigen processing and presentation in BP (Figure 2(a)), endocytic vesicle, ER to Golgi transport vesicle membrane, and MHC class II protein complex in CC (Figure 2(b)), as well as vinculin binding, Ran GTPase binding, and potassium channel regulator activity in MF (Figures 2(c)). Additionally, KEGG pathway enrichment analysis showed that the host genes of the identified DE circRNAs were mainly related to protein processing in the endoplasmic reticulum, other glycan degradation, lysine degradation, homologous recombination, epithelial cell signaling in *Helicobacter pylori* infection, neurotrophin signaling pathways, biosynthesis of nucleotide sugars, aminoacyl-tRNA biosynthesis, endocytosis, and yersinia infection (Figure 3(a)). Based on the number of pathway-related genes, those pathways were sorted as endocytosis, protein processing in the endoplasmic reticulum, yersinia infection, neurotrophin signaling pathways, epithelial cell signaling in *H. pylori* infection, lysine degradation, aminoacyl-tRNA biosynthesis, homologous recombination, biosynthesis of nucleotide sugars, and other glycan degradation (Figure 3(b)).

### 3.3. Validation of RNA Sequencing Using qRT-PCR

**Figure 4:**
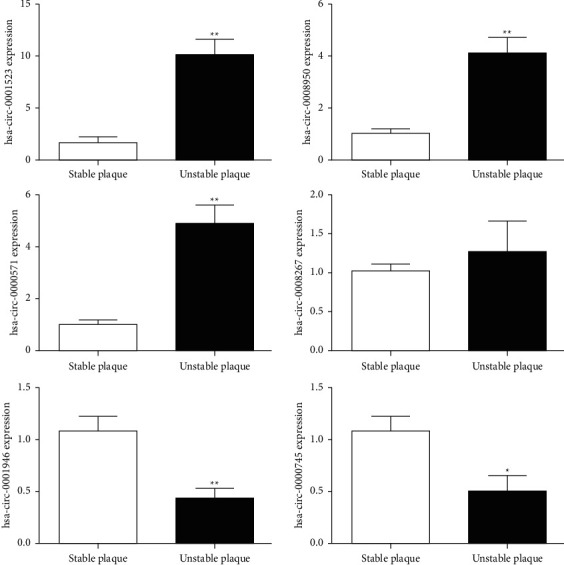
qRT-PCR validation of six DE circRNAs. Four upregulated circRNAs and two downregulated circRNAs from three stable and three unstable plaques are shown. ^*∗*^*P*  <  0.05, ^*∗∗*^*P*  <  0.01 compared with the stable plaques.

Using the qRT-PCR assay, the RNA sequencing results were verified in four upregulated DE circRNAs (hsa-circ-0001523, hsa-circ-0008950, hsa-circ-0000571, and hsa-circ-008267) and two downregulated DE circRNAs (hsa-circ-0001946 and hsa-circ-0000745). The results showed that compared with the stable plaques, the expression levels of hsa-circ-0001523, hsa-circ-0008950, and hsa-circ-0000571 were significantly increased in the unstable plaques (*P*  <  0.05), whereas the expression levels of hsa-circ-0001946 and hsa-circ-0000745 were significantly decreased (*P*  <  0.05, Figure 4), consistent with the expression patterns of the sequencing. For hsa-circ-0008267, there was no significant difference in its expression between the stable and unstable plaques (*P*  >  0.05, Figure 4). The results indicated that the concordance rate of the qRT-PCR results and sequencing data was approximately 83.33%, which implies the relatively high reliability of RNA sequencing.

## 4. Discussion

RNA sequencing supported by biochemical enrichment strategies and in-depth bioinformatic approaches allows comprehensive studies of ncRNAs in the pathogenesis of diseases [6]. The dysregulation of ncRNA expression profiles was found in a different stage of atherosclerosis progression [15]. Nevertheless, a comprehensive understanding of the expression and function of circRNAs in unstable plaques is lacking. This study identified DE circRNAs between unstable and stable plaques and found 344 DE circRNAs in the unstable plaques. Ultimately, the dysregulation of the DE circRNAs was validated in the unstable plaques by qRT-PCR.

CircRNAs have attracted increasing attention for their role and function in the manipulation of multiple metabolic and signaling pathways in the pathogenesis of diseases through their effect on a variety of biological functions, such as their absorbance of microRNAs or proteins and direct control of protein function [16]. The characteristics of the conserved expression of circRNAs and their influence on gene expression indicate that the dysregulation of circRNAs plays an important role in physiological and pathological changes. A previous review summarized the studies that have demonstrated the value of circRNAs as diagnostic biomarkers and therapeutic targets for atherosclerosis [17]. One study by Yu et al. indicated that circRNAs play a role in protecting atherosclerotic plaque stability [5]. The effect of circRNAs on plaque stability indicates a promising direction for the management of plaque rupture. This led us to explore abnormally expressed circRNAs in unstable plaques as compared to that in stable plaques. Our RNA sequencing showed that of a total of 2450 annotated circRNAs, there were 342 upregulated circRNAs and 2 downregulated circRNAs; this is a large volume of data regarding DE circRNAs in unstable plaques. Our RT-qPCR results also showed that hsa-circ-0001523, hsa-circ-0008950, and hsa-circ-0000571 were upregulated, while hsa-circ-0001946 and hsa-circ-0000745 were downregulated in the unstable plaques. Reportedly, some DE circRNAs are important participants in atherosclerotic diseases. For instance, hsa-circ-0001946 was identified in peripheral blood, demonstrating an intimate relationship with coronary atherosclerotic heart disease [18]. A case–control study by Sun et al. found that the correlation of hsa_circ_0001946 to coronary heart disease is independent of other common environmental risk factors [19]. These data indicate the critical role of hsa_circ_0001946 in vascular pathological change; however, the mechanism of action is largely unknown and needs further exploration. Besides hsa_circ_0001946, other DE circRNAs are not functionally understood, especially in the progression of atherosclerosis and plaque stability. Nevertheless, our finding is encouraging, but the function of those dysregulated circRNAs needs further explanation.

We further analyzed the potential effects of the 342 upregulated circRNAs on plaque stability by GO term analysis of their host genes and concluded that the host genes of the upregulated circRNAs are, in the basic pathological processes, indispensable to plaque stability. Remarkably, the GO terms enriched most by upregulated circRNAs in biological process and molecular function were ER to Golgi transport vesicle membrane and Ran GTPase binding. The Golgi apparatus is the biosynthetic center of the secretory pathway and uses vesicle transport to deliver substances to a specific part of the cell or plasma membrane [20]. Golgi is responsible for modifications of the nascent very low-density lipoprotein (VLDL) and the vesicle transport of the VLDL, the precursor for low-density lipoprotein (LDL), to the plasma membrane [21]. A high concentration of plasma LDL cholesterol is one of the critical risk factors that accelerate the progression of atherosclerosis. Golgi vesicle transport plays a role in cholesterol efflux, and with the export of excess cellular cholesterol by cells, Golgi vesicle transport to the plasma membrane increased twofold [22]. Cholesterol in the dense LDL fractions affects carotid plaque cellular composition [23], whereby low LDL cholesterol accompanies a significantly lower prevalence of plaque rupture [24]. Another enriched GO term was Ran GTPase binding, a contributor to GTPase activation. Activated GTPase is generally involved in the pathological process of atherosclerosis [25, 26]. Thus, we deduce that circRNAs may be involved in controlling plaque stability by affecting cholesterol efflux and GTPase activation.

The mechanism of plaque formation and instability is a complex process involving multiple metabolic and signaling pathways [27]. We further performed KEGG analysis to reveal the host genes of significantly upregulated circRNA-related KEGG pathways and found that those dysregulated circRNAs were enriched in protein processing in the endoplasmic reticulum and epithelial cell signaling in *H. pylori* infection. The endoplasmic reticulum is a type of eukaryotic membranous system that provides a venue for some protein processing and lipid synthesis. The function and dysfunction of protein processing in the endoplasmic reticulum play an important role in plaque stability [28, 29]. *H. pylori* infection activates multiple signal transduction pathways that promote inappropriate inflammatory responses and contributes to pathological changes in lipid metabolism and epithelial cell proliferation, survival, and function [30]. Those events associated with *H. pylori* are closely related to plaque formation and stability [31, 32]. Overall, functional analysis by GO and KEGG annotation revealed the important roles of upregulated circRNAs in regulating the plaque stability of the arterial wall. Nevertheless, further studies are warranted to confirm the function of those dysregulated circRNAs in unstable plaques.

Notwithstanding the findings, this study has some limitations. First, the sample size was small, and our findings need to be further confirmed with a larger sample size. Moreover, the specific roles and mechanisms of the identified dysregulated circRNAs in the progression of the unstable plaques should be further investigated *in vitro* and *in vivo*.

In summary, this study identified circRNA expression profiles in stable and unstable plaques and identified plaque-stability-associated dysregulated circRNAs in unstable plaques. Our results provide a comprehensive understanding of circRNAs involved in plaque stability of carotid stenosis and provide a theoretical basis for circRNAs (such as hsa-circ-0001523, hsa-circ-0008950, hsa-circ-0000571, hsa-circ-0001946, and hsa-circ-0000745) to be used as potential targets to prevent or diagnose plaque rupture of carotid stenosis patients in clinical settings.

## Figures and Tables

**Table 1:** Demographic data and chemical detection indicators.

Index	Stable plaque	Unstable plaque
Gender		
Male	1	3
Female	2	0
Age (years, average)	65–85, 73 ± 10.58	61–70, 66.67 ± 4.933
Systolic pressure	152.7 ± 20.03	144 ± 8.386
Diastolic pressure	90.33 ± 12.66	79.67 ± 4.726

**Table 2:** Sequences of all primers for qRT-PCR.

Primers	Sequences (5′-3′)
GAPDH	F: TGACAACTTTGGTATCGTGGAAGG
	R: AGGCAGGGATGATGTTCTGGAGAG

has_circ_0001523	F: AATTTGGAAGGGATCGTG
	R: GGAGGGTAGAATCTGAGGC

hsa_circ_0008950	F: TGTGCCCTCAAATTCCTG
	R: AGTTCCCGTGCCCTTTCC

hsa_circ_0000571	F: CCTCAACAAGTGGGAGAT
	R: GGTTTAGCAGGTGGTTCT

hsa_circ_0008267	F: ACAGCCAGAGGACAACAC
	R: GAGGCATTCTCGACACCC

hsa_circ_0001946	F: CCATCAACTGGCTCAATA
	R: CACAGGTGCCATCGGAAA

hsa_circ_0000745	F: TAAAGGCAAACGGTGAAA
	R: GTGGGAGTGTTGGAAGAAGT

**Table 3:** Top 15 upregulated circRNAs in unstable plaques.

circAltas_ID	log_2_FoldChange	*P* value	Regulation	chr	circBase
hsa-TRAPPC9_0001	10.9394144	4.1*E* − 05	UP	8	hsa_circ_0006904
hsa-TTC39C_0002	10.00991944	8.33*E* − 15	UP	18	hsa_circ_0000839
hsa-MYO15B_0001	9.981122636	5.6*E* − 08	UP	17	—
hsa-P4HB_0002	9.835615642	7.12*E* − 11	UP	17	hsa_circ_0046263
hsa-CTC-435M10_0009	9.728565871	2.44*E* − 09	UP	19	hsa_circ_0051220
hsa-ZNF81_0002	9.477294092	1.18*E* − 08	UP	X	hsa_circ_0006364
hsa-IFI30_0001	9.401377772	2.59*E* − 14	UP	19	hsa_circ_0005571
hsa-RBPMS_0002	9.305309582	2.4*E* − 13	UP	8	hsa_circ_0006539
hsa-PSD3_0018	9.214380058	1.71*E* − 10	UP	8	hsa_circ_0004458
hsa-PSEN1_0011	9.032766078	6.03*E* − 11	UP	14	hsa_circ_0008521
hsa-IBTK_0010	8.907485582	5.39*E* − 10	UP	6	hsa_circ_0002041
hsa-PGS1_0011	8.829415425	2.27*E* − 06	UP	17	hsa_circ_0008410
hsa-SFMBT2_0001	8.770282465	4.74*E* − 09	UP	10	hsa_circ_0017636
hsa-SCARF1_0001	8.696455774	1.4*E* − 08	UP	17	hsa_circ_0000732
hsa-GOLPH3_0002	8.696455774	1.4*E* − 08	UP	5	hsa_circ_0001470

## Data Availability

The data used to support the findings of this study are included within the article.

## Additional Points

(1) There were 344 dysregulated circRNAs in unstable plaques. (2) Most significantly enriched GO terms are Golgi vesicle transport, vacuolar membrane, and GTPase activator. (3) KEGG analysis suggests that DE circRNAs were mainly associated with protein processing in endoplasmic reticulum.
